# Self-perceived disease activity was the strongest predictor of COVID-19 pandemic-related concerns in young people with autoimmune rheumatic diseases, irrespective of their gender, with females reporting higher concerns

**DOI:** 10.1093/rap/rkac031

**Published:** 2022-04-28

**Authors:** Junjie Peng, Puja Mehta, Ayesha Khatun, Wing-Han Wu, Laura Hennelly, Georgia Doolan, Julian R Henty, Paul Howard, Elizabeth Jury, Coziana Ciurtin

**Affiliations:** 1 Centre for Adolescent Rheumatology Versus Arthritis, Department of Medicine; 2 Centre for Inflammation and Tissue Repair, University College London; 3 Department of Clinical Health Psychology, St Mary’s Hospital; 4 Lupus UK; 5 Centre for Rheumatology Research, Department of Medicine, University College London, London, UK

**Keywords:** COVID-19 pandemic–related concern, well-being, resilience, disease activity

## Abstract

**Objectives:**

We report the results of a pilot young patient survey that targeted patients with JSLE and JDM, exploring well-being, resilience and general concern about the coronavirus disease 2019 (COVID-19) pandemic as well as self-assessment of disease activity.

**Methods:**

The survey was completed anonymously by patients who had been approached via the automatically generated hospital database between June and December 2020. In addition to disease characteristics, geographic location, education and employment level, we explored young patients’ resilience, mood and feelings, mental well-being, self-assessed disease activity and general COVID-19 concerns using validated tools and visual analogue scales.

**Results:**

This pilot study found that self-perceived disease activity was the strongest predictor of COVID-19 concern, irrespective of gender, employment and education status or well-being and resilience. Generalized concerns regarding the COVID-19 pandemic were significantly higher in females, although their self-reported DASs were comparable to male respondents.

**Conclusion:**

Our findings highlight a gender bias in the generalized concern related to the COVID-19 pandemic, irrespective of the examined potential confounders. This suggests the need for further research around young patient self-reported outcomes outside hospital visits, especially in the context of gender differences and potential challenges of future pandemics.

Key messages

- Self-perceived disease activity was the strongest predictor of COVID-19 pandemic–related concerns in young people with autoimmune rheumatic diseases, irrespective of their gender.
- Females reported higher COVID-19 pandemic–related concerns.

## Introduction

The impact of the coronavirus disease 2019 (COVID-19) pandemic on the delivery of healthcare for adolescents and young people should not be underestimated, even though this population is less clinically vulnerable to COVID-19 infection. Physiological, psychological and social-role changes during this period heavily influence future well-being and health. The additional consequences of disrupted access to education, work and healthcare, and social and financial stresses during the pandemic have had a significant impact on mental health and well-being [1]. Young people with chronic health conditions already face a disproportionate psychosocial burden compared with their peers and are likely to face additional worries and concerns regarding COVID-19, including heightened health-related anxiety, disrupted routines and reduced access to physical and psychosocial support [2].

As clinicians working in a specialist centre, we aimed to evaluate the impact of the COVID-19 pandemic in adolescents and young adults with JSLE and JDM. These are rare, complex, autoimmune connective tissue disorders with a female preponderance, associated with multiple organ involvement and significant impact on quality of life. Here we report the results of a pilot survey that explored well-being, resilience and concern about the COVID-19 pandemic in patients with JSLE and JDM.

## Methods

The survey was completed anonymously by patients between June and December 2020. The hospital digital database generated a complete list of patients with JDM and JSLE who were under the care of the Department for Adolescent Rheumatology at University College London Hospital in 2020. Automatic e-mails were sent to registered patient e-mail addresses available in the database and patients ≥16 years of age (who were legally able to consent at the time of inclusion in this exploratory survey) were invited to complete the survey anonymously by clicking on a link to a RedCap questionnaire. To minimize the selection bias, none of the patients were approached to complete the questionnaire when attending clinics in person. The survey was approved by the Health Research Authority (HRA) committee for England and Wales (ref. 20/HRA/2565) on 20 May 2020.

Patients provided electronic consent before proceeding with completing the survey online, as required by the HRA approval. Results were analysed using descriptive statistics (using R version 4.1.3.; R Foundation for Statistical Computing, Vienna, Austria). The survey (Supplementary Data S1, available at *Rheumatology Advances in Practice* online) contained questions about the patients’ demographics (age, ethnicity, gender), employment and education status, disease duration, age at onset, as well as well-being or resilience using the validated Mood and Feeling Questionnaire (MFQ) [3], resilience scale [4] and the Warwick–Edinburgh Mental Wellbeing Scale (WEMWBS) [5]. The patients were also required to complete a visual analogue scale (VAS; 0–100) to rate their general concerns regarding the general impact of COVID-19 on their lives (0, no concern; 100, extremely concerned), as well as self-assess their global disease activity (0, no disease activity; 100, the most active disease imaginable). Data were analysed using descriptive statistics (Fisher’s exact test, normality test, Welch’s t-test, Mann–Whitney U test) and linear regression (R).

## Results

**Fig. 1:**
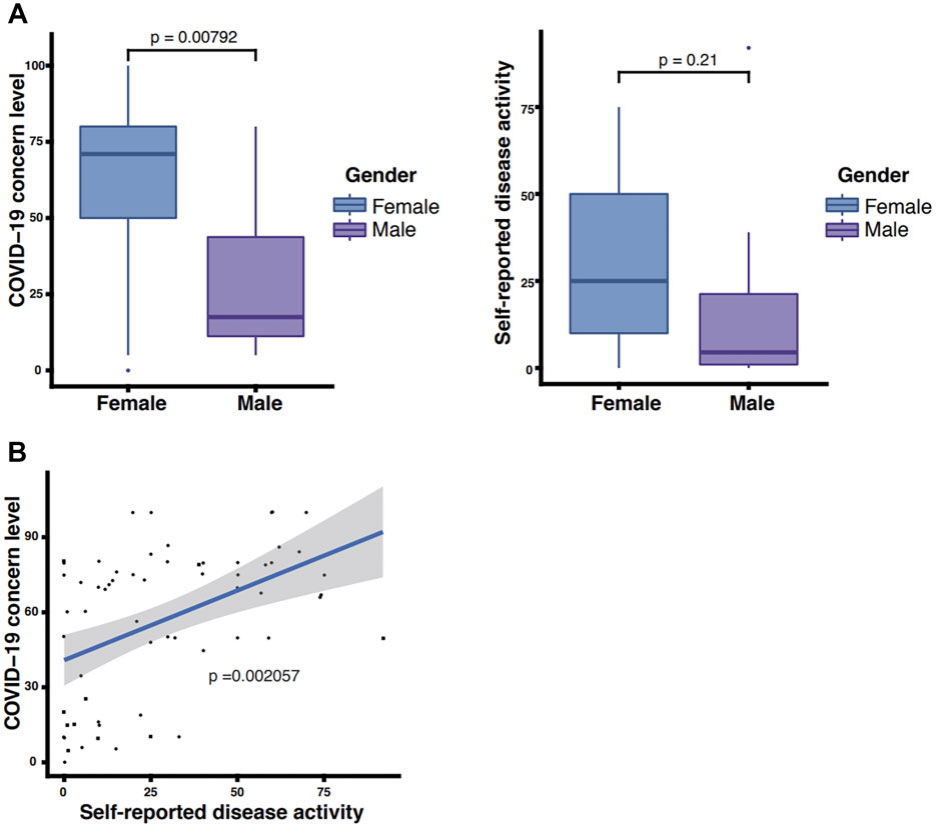
Determinants of COVID-19 concern level and self-perceived disease activity **(A)** First panel—Differences in COVID concern level (VAS 1–100; 100 being the highest concern regarding health outcomes during the COVID-19 pandemic) according to gender in patients with JSLE and JDM. Second panel—Differences in self-reported disease activity (VAS 1–100; 100 being the highest self-perceived disease activity) according to gender in patients with JSLE and JDM. **(B)** Linear regression analysis adjusted for age, ethnicity and gender demonstrated that self-reported disease activity (using a global VAS) was the strongest determinant of concerns associated with the COVID-19 pandemic.

There were 63 respondents (53 females, 10 males) who answered the survey during the recruitment period (47 respondents had JSLE and 16 had JDM, which reflects the patient split in our hospital cohort). The survey had a 47% response rate, which is not unsurprising considering that patients were not approached face to face, as many of the routine clinical assessments were conducted virtually at the peak of the COVID-19 pandemic. Reflecting the significant gender bias in JSLE and the higher prevalence of JSLE compared with JDM, there was a predominance of young females completing the survey (84%). There were no significant gender differences in the disease duration, age at diagnosis, age of responders or immunosuppressive medication according to self-reported gender (Table 1). The majority of patients were living outside London, as we are a tertiary centre for adolescent and young adult rheumatology. Generalized concerns regarding the COVID-19 pandemic were significantly higher in females (*P* = 0.007), although their self-reported disease activity scores were comparable to those of male respondents (*P* = 0.205) (Table 1, Fig. 1A). The self-reported resilience was almost identical in young men and women (*P* = 0.99).

**Table 1:** Patient characteristics according to self-reported gender

Characteristics	Female	Male	*P*-value
Patients, *n*	53	10	–
Current age, years, mean (IQR)	22.42 (19.00–27.00)	22.86 (17.15–29.25)	0.87
Age at diagnosis, years, mean (IQR)	11.47 (9.00–14.00)	10.00 (8.00–11.75)	0.26
Disease duration, months, mean (IQR)	10.94 (5.00–16.00)	12.86 (9.70–18.75)	0.46
Ethnicity, *n* (%)			
White	33 (62.3)	8 (80.0)	0.47
Non-white	20 (37.7)	2 (20.0)	
Location, *n* (%)			
London	13 (24.5)	2 (20.0)	1.00
Other	40 (75.5)	8 (80.0)	
Immunosuppressive treatment, *n* (%)	40 (75.4)	8 (80)	0.97
Prednisolone treatment, *n* (%)	22 (41.5)	5 (50)	0.83
Well-being (VAS 0–5), mean (IQR)	3.066 (2.500–3.500)	3.486 (3.304–4.143)	0.27
Resilience (VAS 0–7), mean (IQR)	4.764 (3.714–5.571)	4.757 (3.929–6.161)	0.99
Self-reported disease activity (VAS 0–100), mean (IQR)	30.83 (10.00–50.00)	17.70 (1.00–21.25)	0.205
Self-reported COVID concern (VAS 0–100), mean (IQR)	61.77 (50.00–80.00)	30.90 (11.25–43.75)	0.007
Still in school, *n* (%)			
No	27 (50.9)	5 (50.0)	1.00
Yes	26 (49.1)	5 (50.0)	
Of those previously employed, currently working, *n* (%)			
No	12 (44.4)	0 (0)	0.24
Yes	15 (55.6)	4 (100)	

IQR: interquartile range.

Linear regression analysis, adjusted for age, ethnicity and gender, demonstrated that self-reported disease activity (using a global VAS) was the strongest determinant of concerns associated with the COVID-19 pandemic (*P* < 0.0003) (Fig. 1B), which was evident in both females (*P* = 0.003) and males (*P* = 0.004). Longer disease duration was associated with general concerns regarding the COVID-19 pandemic in males only (*P* = 0.018). There was no association between COVID-19-associated concerns and employment and education status, or well-being or resilience, using the validated MFQ [3], resilience scale [4] and the WEMWBS [5].

## Discussion

To our knowledge, this is the first evaluation of the impact of the COVID-19 pandemic on patients with JSLE and JDM from a patient rather than a parent/caregiver perspective [6] and focussed on self-reported psychological and disease-related outcomes rather than on the quality of care received during the pandemic as in previous reports in young patients with rheumatic diseases [6, 7]. We found that self-perceived disease activity was the strongest predictor of COVID-19 pandemic–related concern, irrespective of gender.

Our results demonstrate that gender differences should be considered when assessing patients and that concerns regarding COVID-19 strongly correlate with patient-reported disease activity when accounting for other confounders. The mental health/psychology outcomes we investigated in this pilot study (self-reported mood and feelings, well-being and perceived resilience) did not have a significant impact on COVID-19-associated concerns, which was also the case with the respondents’ educational or employment status. The results of this study suggest that having a chronic condition as a young person was more relevant than the other factors investigated here in determining the self-reported level of concern related to the pandemic, which is an important message for public health.

We were unable to compare patient-reported and physician-assessed disease activity at the same time point, given restricted access to clinic appointments during the height of the pandemic. During the period captured by this pilot survey, the majority of patients were assessed remotely via telephone consultations, with only a small proportion (∼10–15%) being assessed in person if identified as potentially flaring. This survey did not explore possible associations between clinical outcomes at the last hospital visit and the participants’ responses, as this was beyond the scope of this study.

Previous psychology research has identified gender differences in well-being in healthy young people [8], while the most important predictors of well-being in emerging adulthood were higher self-confidence and lower negative self-evaluation [9], aspects that are likely to be affected by having to live with a chronic condition such as JSLE or JDM. In addition, well-being at the age of emerging adulthood is recognized as having a significant impact on further psychological well-being [10]; therefore, addressing additional psychological stressors, such as general concern related to the COVID-19 pandemic, in young people with chronic conditions could have long-term benefits.

The main limitations of this exploratory survey are related to the small sample size and single-centre and cross-sectional study design, which do not allow for generalization of results, as well as an inability to establish any causal relationship between the outcomes measured. Not surprisingly, only 15% of respondents were males, which reflects the gender bias reported by larger JSLE cohorts (e.g. female:male ratio is 6:1 in the UK JSLE cohort [11]). As the prevalence of JDM is 10 times lower that of than JSLE, as expected, the survey captured fewer JDM patients. There are still uncertainties about the outcomes of COVID-19 infection/vaccination in children and young people with chronic inflammatory conditions, despite the reassuring signals to date [12–15]. At the time of this survey data collection, COVID-19 vaccines were not licenced for use in the UK, therefore no other protective interventions, apart from self-isolation and social distancing, were available during the first wave of the pandemic. This is likely to be reflected in the general COVID-19-associated concern reported by young patients. Although we did not capture detailed information regarding potential neurological manifestations associated with patients’ underlying conditions, we do not expect that severely ill young patients would have taken the time to complete this online survey. As mentioned above, the survey was not disseminated to patients attending hospital appointments, to minimize the selection bias in favour of patients with more severe disease at the time of survey completion.

Further research to understand gender differences in self-reported outcomes of chronic inflammatory conditions, as well as general concerns about health, is required to enable targeted approaches to protect young peoples’ well-being, especially when their access to direct care is restricted. Understanding the relationship between objective disease assessments and young people’s self-reported outcomes can also contribute to improved strategies to preserve patients’ quality of life in the context of various challenges associated with future potential pandemics.

## Supplementary Material

rkac031_Supplementary_Data
