# Supplementary material for: Self-perceived disease activity was the strongest predictor of COVID-19 pandemic-related concerns in young people with autoimmune rheumatic diseases, irrespective of their gender, with females reporting higher concerns
Source: Rheumatol Adv Pract. 2022 Apr 28;6(2):rkac031. doi: 10.1093/rap/rkac031 (PMC9113467; doi:10.1093/rap/rkac031)
Supplement: rkac031_Supplementary_Data [file rkac031_supplementary_data.pdf]

Impact of COVID-19 on patients with JDM and JSLE

This study aims to investigate the impact of the COVID-19 pandemic on the wellbeing of patient's suffering with juvenile dermatomyositis (JDM) and juvenile lupus (JSLE). It will assess the resources and coping strategies used, and identify mechanisms for improving access to resources for patients affected with these diagnoses.

If you are interested please help us by filling in the survey below

Participation is voluntary and you can withdraw at any time by withholding your questionnaire and not submitting. The survey is anonymous and the research team will not have access to your personal data. Where free text is available please do not include any personal information that could potentially be used to identify you. The study was complied with our sponsor's University College London hospital data protection policy. All data is anonymised and will be kept in UCLH data safe haven. For other further queries in regards to data or the study please contact; uclh.ucl-rheumaya@nhs.net

---

Are you completing this questionnaire on behalf of yourself?

- ◯ Yes
- ◯ No

If not, what is your relationship with the patient?

____
(Please complete the questions below as responding on behalf on the young patient with JSLE/JDM.)

What is your age?

____

What is your diagnosis?

____

At what age have you been diagnosed?

____

What is your ethnic background?

- ◯ English / Welsh / Scottish / Northern Irish / British Irish
- ◯ Gypsy or Irish Traveller
- ◯ Any other White background
- ◯ White and Black Caribbean
- ◯ White and Black African
- ◯ White and Asian
- ◯ Asian / Asian British
- ◯ Indian
- ◯ Pakistani
- ◯ Bangladeshi
- ◯ Chinese
- ◯ Any other Asian background
- ◯ Black / African / Caribbean / Black British
- ◯ African
- ◯ Caribbean
- ◯ Any other Black / African / Caribbean background
- ◯ Arab
- ◯ Any other ethnic group

Which village, town or city do you currently live in?

____

Are you Male/Female or other?

- ◯ Male
- ◯ Female
- ◯ Other

If other, please specify	__________________________
On a scale of 1-100 how active would you consider your disease at present? (1=not active, 100=very active).	Not active ... Very active<br>(Place a mark on the scale above)
Have you been prescribed any medication for your lupus?	◯ Yes<br>◯ No
If yes, which ones?	☐ Steroids tablets<br>☐ Steroids drip in the last month<br>☐ Methotrexate<br>☐ Hydroxychloroquine<br>☐ Mycophenolate mofetil<br>☐ Azathioprine<br>☐ Cyclophosphamide<br>☐ Rituximab<br>☐ Other<br>(Please select all that apply)
If other, please specify	__________________________
Are you taking any medication for your lupus? yes/no?	◯ Yes<br>◯ No
If yes, which ones?	☐ Steroids tablets<br>☐ Steroid drip in the last month<br>☐ Methotrexate<br>☐ Hydroxychloroquine<br>☐ Mycophenolate mofetil<br>☐ Azathioprine<br>☐ Cyclophosphamide<br>☐ Rituximab<br>☐ Other<br>(Please select all that apply)
If other, please specify	__________________________
If you are currently not taking any medication, why?	☐ Doctor's recommendation as my lupus is currently in remission<br>☐ I decided to stop my medications because of concerns about COVID-19 infection risk<br>☐ My GP/ doctor stopped my medications because of concerns about COVID-19 infection risk<br>☐ Supply issues<br>☐ Other<br>(Please select all that apply)
If other, please specify	__________________________
Have you had a rheumatology telephone/video consultation since the COVID-19 pandemic started?	◯ Yes<br>◯ No

Did you find the telephone/video consultation helpful?	◯ Yes<br>◯ No<br>◯ Not applicable
Was it as helpful as an appointment you attended before the COVID-19 lockdown?	◯ Yes<br>◯ No<br>◯ Not applicable
Have you been admitted to hospital due to lupus in the last 3 months?	◯ Yes<br>◯ No

Concern about COVID-19 in general

On a scale of 1-100 how concerned are you about COVID-19 in general? (1 = no concern, 100 very concerned)

No concern — Very concerned

(Place a mark on the scale above)

Do you have any other comments relating to COVID-19 concern in general?

____

Have you received any information about the degrees of isolation?

◯ Yes ◯ No

Have you had information from (please tick all that apply)

☐ NHS website ☐ Watching the news ☐ Public Health England/Wales/ Scotland website, as appropriate ☐ Consultant ☐ GP ☐ Clinical Nurse Specialist (CNS) ☐ Other health care workers ☐ Charities/Patient organisations (e.g. Lupus UK, Versus Arthritis)
(Please select all that apply)

How did you receive this information?

☐ A letter ☐ Via telephone/video consultation ☐ A text message ☐ Through your hospital records portal if you have access to your hospital file ☐ Hospital Website ☐ Public Health England/Wales/ Scotland website, as appropriate ☐ NHS website ☐ Other
(Please select all that apply)

If other, please specify

____

What type of isolation have you been advised to adhere to?

◯ Self-isolation and strict social-distancing ◯ Shielding ◯ Received no advice at all

Anxiety about COVID-19 in your household

On a scale of 1-100 how worried are you about COVID-19 in your household? (1 = not worried, 100 very worried)

Not worried — Very worried

(Place a mark on the scale above)

Has a member of your household had COVID-19 known symptoms?

☐ Yourself ☐ Your parent/guardian (if appropriate) ☐ Your child/children (if appropriate) ☐ Any other member of the household (including siblings who live with you) ☐ Other ☐ Not applicable

Has a member of your household tested positive for COVID-19?

☐ Your parent/guardian (if appropriate)
☐ Your child/children (if appropriate)
☐ Any other member of the household (including siblings who live with you)
☐ Other ☐ Not applicable

If you are a young person living with your parents, has the COVID-19 lockdown affected your usual household living arrangements?

○ Yes ○ No ○ Not applicable

If so, how?

☐ More family members staying in the family house? ☐ If parents or your partner (as appropriate) live separately, not being able to share the time between both parents
☐ Living arrangements have not been affected with lockdown ☐ Not applicable

If you are a young adult and have children, has the COVID lockdown affected your usual household living arrangements?

○ Yes ○ No ○ Not applicable

If so, how?

☐ More family members staying in the house
☐ If parents or your partner (as appropriate) live separately, not being able to share looking after your children between both parents
☐ Living arrangements have not been affected by lockdown ☐ Not applicable

Exercise

Does your household have a garden?	◯ Yes ◯ No
If you do have a garden can you access it?	◯ Yes ◯ No
Are you within walking distance to a park?	◯ Yes ◯ No
What form of exercise do you take part in?	☐ Walking ☐ Running<br>☐ Cycling ☐ In door exercises<br>☐ Other<br>(Please select all that apply)
If other, please specify	____________________________________
If you are shielding, are you able to undertake daily exercise at home?	◯ Yes ◯ No ◯ Not applicable
If shielding, can you give examples of the types of activities or approaches you have taken? Any other comments?	____________________________________
If self-isolating and strictly adhering to social distancing, are you able to undertake daily exercise at home?	◯ Yes ◯ No ◯ Not applicable
Can you give examples of the types of activities or approaches you have taken during self isolation? Any other comments?	____________________________________
Do you undertake physiotherapy at home (using written information/video training courses)?	◯ Yes ◯ No ◯ Not applicable
Are you able to undertake physiotherapy exercises at home?	◯ Yes ◯ No ◯ Not applicable
Are you able to communicate with your physiotherapist? Any other comments?	____________________________________

Mental Well-being

If you are feeling low, what strategies and resources do you generally employ to make yourself feel better?	☐ Exercise ☐ Spending time talking with friends or family ☐ Listening to music or watching television alone ☐ Listening to music/watching screens with family or online with friends ☐ Screen time which includes face to face interaction e.g. skype, zoom or face time with friends or family ☐ Reading a book ☐ Listening to a meditation/well-being app ☐ Spending time with pets ☐ Nature walks in the park ☐ Spending time helping others ☐ Other (Please select all that apply)
If other, please specify	__________________________________
What do you do now to improve your mental well-being that you did not do before the lock-down?	☐ Exercise ☐ Spending time talking with friends or family ☐ Listening to music or watching television alone ☐ Listening to music/watching screens with family or online with friends ☐ Screen time which includes face to face interaction e.g skype, zoom or face time with friends or family ☐ Reading a book ☐ Listening to a meditation/well-being app ☐ Spending time with pets ☐ Nature walks in the park ☐ Spending time helping others ☐ Other (Please select all that apply)
If other, please specify	__________________________________
How does your mental well-being compare to prior the lock-down?	○ The same ○ Slightly Better ○ Much better ○ Slightly Worse ○ Much worse
How is your family staying positive or keeping the morale high in the household?	○ Doing more physical activities such as trips to the park; ○ Board games with family ○ Gaming (playing computer games) ○ Being allowed to watch more TV or use of the computer ○ Sharing schoolwork ○ Sharing and helping out with household tasks ○ Spending more time with each other as a family. ○ Other (Please select all that apply)
If other, please specify	__________________________________
Have you accessed any mental health professional or specialist help?	○ Yes ○ No ○ I don't feel I need it

If yes, what type of mental health professional/specialist help?	☐ Phone/video consultations with psychologist ☐ Talking with your GP<br>☐ Talking with your rheumatologist<br>☐ Talking with a nurse<br>☐ Websites offering mental health advice<br>☐ Not applicable ☐ Other
If other, please specify	__________________________
Have you accessed non-specialist help? For example a focussed chat with a family or friend?	◯ Yes ◯ No ◯ Not applicable<br>◯ Other
If other, please specify	__________________________
If other type of help, please specify:	__________________________
Would you be willing to contribute to creating resources for other people who have a similar diagnosis to you (e.g. sharing your experience or providing advice through a video/ blog, contributing to writing a patient support information leaflet?	◯ Yes ◯ No
Is there anything you would like to do to increase your physical or mental well-being that you are currently unable to?	◯ Yes ◯ No
If yes, please specify	__________________________
What resource do you wish you had that you do not currently have access to?	☐ Online support forum with people with the same diagnosis as me;<br>☐ Online blogs/videos of coping mechanisms<br>☐ Other
If other, please specify	__________________________

Education

Are you still in education?	◯ Yes ◯ No
During your home schooling have you been given access to virtual learning?	◯ Yes ◯ No ◯ Not applicable
During your home schooling have you been given homework?	◯ Yes ◯ No ◯ Not applicable
During your home schooling, have you been receiving support from school?	◯ Yes ◯ No ◯ Not applicable
If so, what forms of educational support have you received?	◯ Access to educational videos/books<br>◯ Participation in virtual classrooms;<br>◯ Participation in virtual group activities.<br>◯ Other
If other, please specify:	__________________________________
On a scale of 1-100, how concerned are you about the effects of the COVID-19 pandemic on your education? (1=no concern, 100= Very concerned)	No concern ... Very concerned<br>*(Place a mark on the scale above)*
On a scale of 1-100 how concerned are you about the effects of the COVID-19 pandemic on your future job prospects? (1=no concern, 100= Very concerned)	No concern ... Very concerned<br>*(Place a mark on the scale above)*

Work

Have you been employed before the pandemic started? ◯ Yes ◯ No

Are you currently working? ◯ Yes ◯ No

If you are not working or in education, on a scale of 1-100 how concerned are you about the effects of the COVID-19 pandemic on your future job prospects? (1=no concern, 100= Very concerned)

No concern — Very concerned

(Place a mark on the scale above)

On a scale of 1-100 how concerned are you about the effects of the COVID-19 pandemic on your work life? (1=no concern, 100= Very concerned)

No concern — Very concerned

(Place a mark on the scale above)

On a scale of 1-100 how concerned are you about the effects of the COVID-19 pandemic on your future career? (1=no concern, 100= Very concerned)

No concern — Very concerned

(Place a mark on the scale above)

On a scale of 1-100, how supportive do you feel your employer has been towards you given your diagnosis of JDM/JSLE and in light of COVID-19 pandemic? (1 = not supportive, 100 = very supportive).

Not supportive — Very supportive

(Place a mark on the scale above)

Are you worried about maintaining your finances and income? ◯ Yes ◯ No

If you have been working before the lock-down, have you been allowed to work from home? ◯ Yes ◯ No

Are you able to work from home? ◯ Yes ◯ No

Are you a key worker and still going to work? ◯ Yes ◯ No

How you feel RIGHT NOW

Please tick the box that best describes how you feel right now

		Not at all	Somewhat	Moderately so	Very much so
1	I feel calm	☐	☐	☐	☐
2	I feel secure	☐	☐	☐	☐
3	I am tense	☐	☐	☐	☐
4	I feel strained	☐	☐	☐	☐
5	I feel at ease	☐	☐	☐	☐
6	I feel upset	☐	☐	☐	☐
7	I am presently worrying over possible misfortunes	☐	☐	☐	☐
8	I feel satisfied	☐	☐	☐	☐
9	I feel frightened	☐	☐	☐	☐
10	I feel comfortable	☐	☐	☐	☐
11	I feel self-confident	☐	☐	☐	☐
12	I feel nervous	☐	☐	☐	☐
13	I am jittery	☐	☐	☐	☐
14	I feel indecisive	☐	☐	☐	☐
15	I am relaxed	☐	☐	☐	☐
16	I feel content	☐	☐	☐	☐
17	I am worried	☐	☐	☐	☐
18	I feel confused	☐	☐	☐	☐
19	I feel steady	☐	☐	☐	☐
20	I feel pleasant	☐	☐	☐	☐

Well-being

Please tick the box that best describes your experience of each over the last two weeks

		None of the time	Rarely	Some of the time	Often	All of the time
1	I've been feeling optimistic about the future	☐	☐	☐	☐	☐
2	I've been feeling useful	☐	☐	☐	☐	☐
3	I've been feeling relaxed	☐	☐	☐	☐	☐
4	I've been feeling interested in other people	☐	☐	☐	☐	☐
5	I've had energy to spare	☐	☐	☐	☐	☐
6	I've been dealing with problems well	☐	☐	☐	☐	☐
7	I've been thinking clearly	☐	☐	☐	☐	☐
8	I've been feeling good about myself	☐	☐	☐	☐	☐
9	I've been feeling close to other people	☐	☐	☐	☐	☐
10	I've been feeling confident	☐	☐	☐	☐	☐
11	I've been able to make up my own mind about things	☐	☐	☐	☐	☐
12	I've been feeling loved	☐	☐	☐	☐	☐
13	I've been interested in new things	☐	☐	☐	☐	☐
14	I've been feeling cheerful	☐	☐	☐	☐	☐

Resilience

Please read each statement and tick the box to the right of each statement that best indicates your feelings about the statement.

		1 Strongly disagree	2,	3	4	5	6	7 Strongly agree
1	I usually manage one way or another	☐	☐	☐	☐	☐	☐	☐
2	I feel proud that I have accomplished things in my life	☐	☐	☐	☐	☐	☐	☐
3	I usually take things in my stride	☐	☐	☐	☐	☐	☐	☐
4	I am friends with myself	☐	☐	☐	☐	☐	☐	☐
5	I feel that I can handle many things at a time	☐	☐	☐	☐	☐	☐	☐
6	I am determined	☐	☐	☐	☐	☐	☐	☐
7	I can get through difficult times because I have experienced difficulty before	☐	☐	☐	☐	☐	☐	☐
8	I have self-discipline	☐	☐	☐	☐	☐	☐	☐
9	I keep thing interested	☐	☐	☐	☐	☐	☐	☐
10	I can usually find something to laugh about	☐	☐	☐	☐	☐	☐	☐
11	My belief in myself gets me through hard times	☐	☐	☐	☐	☐	☐	☐
12	In an emergency I am someone people can generally rely on	☐	☐	☐	☐	☐	☐	☐
13	My life has meaning	☐	☐	☐	☐	☐	☐	☐
14	When I am in a difficult situation, I can usually find my way out of it	☐	☐	☐	☐	☐	☐	☐

If you or the young person you are caring for experiences low mood, depression or feeling that their life has no meaning, please contact your/their GP to discuss your symptoms.

For practical tips, please visit:
https://www.versusarthritis.org/news/2019/june/arthritis-and-depression-what-you-can-do-about-it/

https://www.gov.uk/government/publications/covid-19-guidance-for-the-public-on-mental-health-and-wellbeing
